# Model of phenotypic evolution in hermaphroditic populations

**DOI:** 10.1007/s00285-014-0798-3

**Published:** 2014-05-16

**Authors:** Ryszard Rudnicki, Paweł Zwoleński

**Affiliations:** Institute of Mathematics, Polish Academy of Sciences, Bankowa 14, 40-007 Katowice, Poland

**Keywords:** Measure valued process, Phenotypic evolution, Sexual population model, Nonlinear transport equation, Asymptotic stability, Primary 47J35, Secondary 34G20, 60K35, 92D15

## Abstract

We consider an individual based model of phenotypic evolution in hermaphroditic populations which includes random and assortative mating of individuals. By increasing the number of individuals to infinity we obtain a nonlinear transport equation, which describes the evolution of phenotypic distribution. The main result of the paper is a theorem on asymptotic stability of trait distribution. This theorem is applied to models with the offspring trait distribution given by additive and multiplicative random perturbations of the parental mean trait.

## Introduction

This paper studies the evolution of phenotypic traits in hermaphroditic populations, i.e. populations where every individual has both male and female reproductive system. A great part of these populations has formed various defense mechanisms against self-fertilization (*autogamy*) to guarantee genetic diversification (e.g. a proper shape of a flower can inhibit self-pollination in some species of plants). In that case, individuals can only mate with others to copulate and cross-fertilize. Nonetheless, cross-fertilisation occurs in some hermaphroditic species. Hermaphroditic populations are plentiful among both water and terrestrial animals as well as plants. Some of examples include Sponge (*Porifera*), *Turbelleria*, Cestoda (*Cestoidea*), *Lumbricidae*, some of mollusks such as sea slug Blue Dragon (*Glaucus atlanticus*) and various kinds of land snails or majority of flowering plants (angiosperms).

A considerable amount of literature has been published on modelling asexual populations by means of microscopic description of trait evolution. Macroscopic approximations of that models were derived in the forms of deterministic processes or superprocesses (see Champagnat 2006; Champagnat et al. 2008; Fournier and Méléard 2004; Ferrière and Tran 2009; Méléard and Tran 2009). In this paper we formulate an individual based model to describe the phenotypic evolution in hermaphroditic populations. We consider a large population of small individuals characterized by their traits. The traits are assumed to be unchanged during lifetime, and their examples include skin colour, the shape of a leaf and shell pattern. All the individuals are capable of mating or self-fertilizing to give birth to an offspring.

We consider a general model of mating, which includes both random and assortative mating. The first particular case is a semi-random mating model. This model is based on the assumption that each individual has an initial capability of mating depending on its trait. This mating model is similar to models describing aggregation processes in phytoplankton dynamics (see Arino and Rudnicki 2004; Rudnicki and Wieczorek 2006a, b). The second particular case is an assortative mating model. In this model, the individuals with similar traits mate more often than they would choose a partner randomly. We adapt a model based on a preference function Doebeli et al. (2007), Gavrilets and Boake (1998), Matessi et al. (2001), Polechová and Barton (2005), Schneider and Bürger (2006), Schneider and Peischl (2011), usually used in two-sex populations, to the hermaphrodites. The consequence of mating or self-fertilization is a birth of a new individual. The trait of this individual is given by a random variable that depends only on traits of the parents. Each individual can die naturally or when competing with others. We consider a continuous time model, and we assume that all above-mentioned events happen randomly.

The model presented in this paper is a hermaphroditic analogue of the asexual model introduced by Bolker and Pacala (1997), Law and Dieckmann (2002) and studied by Fournier and Méléard (2004). Despite the vast literature concerning individual based models and their macroscopic approximations, only a few models involving mating processes have appeared so far (Collet et al. 2013; Remenik 2009).

One of our aims is to study a macroscopic deterministic approximation of the model. We obtain it by increasing the number of individuals in the population to infinity, with simultaneous decrease in the mass of each individual. After suitable scaling of parameters, the limit passage leads to an integro-differential equation. Solutions of the equation describe the evolution of trait distribution. We also study the existence and uniqueness of the solutions. We investigate extinction and persistence of the population and convergence of its size to some stable level.

The main aim of our paper is to prove asymptotic stability of trait distribution. Asymptotic behavior of the solutions is characterized by conservation of mean phenotypic trait. We apply our main theorem to two specific models. In these models the offspring trait is the parental mean trait randomly perturbed by some external environmental effects or genetic mutations. In the first model, the noise is additive. The property of additivity allows us to derive a formula for the stationary phenotypic distribution. The second model contains multiplicative noise, and it includes, as a special case, the Tjon–Wu version of the classic Boltzmann equation (see Bobylev 1976; Krook and Wu 1977; Tjon and Wu 1979). The Tjon–Wu equation describes the distribution of energy of particles. As a by-product of our investigation we give a simple proof of the theorem of Lasota and Traple (see Lasota 2002; Lasota and Traple 1999) concerning asymptotic stability of this equation. Addtionally, an example of the trait reduction is given. In this case, in a long period of time, all traits reduce to a particular one, which is the mean trait of the initial population.

The scheme of the paper is following. In Sect. 2 we collect all assumptions on the dynamics of the population. In Sect. 3 we introduce a stochastic process corresponding to our individual based model. Section 4 is devoted to the macroscopic approximation, the limiting equation and its solutions. In particular, we give results about extinction and persistence of the population and stabilization of its size. In Sect. 5 we formulate the results concerning the asymptotic stability, and we give examples of their applications. Finally, in the last section we discuss problems for future investigation concerning assortative mating models.

## Individual-based model

Let us fix a positive integer $d$. We assume that every individual is described by a phenotypic trait $x$, which belongs to some closed and convex subset $F$ of ${\mathbb {R}}^d$, whose interior is nonempty. The trait of an individual does not change during its lifetime.

### Random mating

In sexually reproducing populations a mating process highly depends on a given species. We will consider both random and assortative mating. In classical genetics individuals mate randomly—the choice of partner is not influenced by the traits (*panmixia*). Random mating occurs often in plants, but it is also observed in some hermaphroditic animals (Baur 1992). We study a semi-random mating model in which the mating rate depends on the trait. An individual described by the trait $x$ is capable of mating/self-fertilizing with rate $p(x)$, where $p$ is a positive function of the trait.

Consider a population which consists of $n$ individuals with traits $x_1,\dots ,x_n$. Since two different individuals may have the same trait, it is useful to describe the state of the population as the multiset

$$\begin{aligned} {\mathbf {x}}=\{x_1,\dots ,x_n\}. \end{aligned}$$

We recall that a *multiset* (or *bag*) is a generalization of the notion of a set in which the members are allowed to appear more than once. We suppose that any individual can mate with an individual of trait $x_j$ with the following probability

$$\begin{aligned} \frac{p(x_j)}{\sum \nolimits _{l=1}^n p(x_l)}. \end{aligned}$$

Thus the mating rate of individuals with traits $x_i$ and $x_j$ is given by

1 $$\begin{aligned} m(x_i,x_j;{\mathbf {x}})=\frac{p(x_i)p(x_j)}{\sum \nolimits _{l=1}^n p(x_l)}. \end{aligned}$$

The figure $m(x_i,x_i;{\mathbf {x}})$ is a self-fertilization rate. In the case of populations without self-fertilization we assume that

2 $$\begin{aligned} m(x_i,x_j;{\mathbf {x}})=\frac{p(x_i)p(x_j)}{2}\left( \frac{1}{\sum \nolimits _{l\ne i} p(x_l)}+\frac{1}{\sum \nolimits _{l\ne j} p(x_l)}\right) \end{aligned}$$

if $i\ne j$ and $m(x_i,x_i;{\mathbf {x}})=0$. Let us observe that in both cases the mating rate is a symmetric function of $x_i$ and $x_j$ but only in the first case we have $\sum _{j=1}^{n}m(x_i,x_j;{\mathbf {x}})=p(x_i)$. If we pass with the number of individuals to infinity, and replace the discrete model by the infinitesimal model with trait distribution described by a continuous measure $\mu$, then the mating rate in both cases is given by

3 $$\begin{aligned} m(x,y;\mu )=\frac{p(x)p(y)}{\int \nolimits _F p(z)\mu (dz)}. \end{aligned}$$

### Assortative mating

Now we consider models with assortative mating, i.e. when individuals of the similar traits mate more often than they choose a partner randomly. Assortative mating can be modelled in different ways. For example one can use matching theory, according to that, each participant ranks all the potential partners according to its preferences, and attempts to pair with the highest-ranking one (Almeida and de Abreu 2003; Puebla et al. 2012). Such models are very interesting but difficult to analyze. The most popular models of assortative mating are based on the assumption that a random encounter between two individuals with traits $x$ and $y$ depends on a preference function $a(x,y)$ (Doebeli et al. 2007; Gavrilets and Boake 1998; Matessi et al. 2001; Polechová and Barton 2005; Schneider and Bürger 2006; Schneider and Peischl 2011). We consider only the case when all the individuals have the same initial capability of mating $p(x)=1$.

Usually, it is assumed that $a(x,y)=\varphi (\Vert x-y\Vert )$, where $\varphi : [0,\infty )\rightarrow [0,\infty )$ is a continuous and decreasing function. It means that if the population consists of $n$ members with traits $x_1,\dots ,x_n$, then individuals of traits $x_i$, $x_j$ mate with rate

4 $$\begin{aligned} m(x_i,x_j;{\mathbf {x}})=\frac{a(x_i,x_j)}{\sum \nolimits _{l=1}^n a(x_i,x_l)}=\frac{\varphi (\Vert x_i-x_j\Vert )}{\sum \nolimits _{l=1}^n \varphi (\Vert x_i-x_l\Vert )}. \end{aligned}$$

Note that in general, the function $m$ is not symmetric in $x_i$ and $x_j$, and usually it describes mating in two-sex populations. Then the first argument in $m$ refers to a female. Females are assumed to mate only once, whereas males may participate in multiple matings. We have $\sum _{j=1}^n m(x_i,x_j;{\mathbf {x}})=1$ for each $i$, which means that all females mate with the same rate. The mating rate in the infinitesimal model is of the form

5 $$\begin{aligned} m(x,y;\mu )=\frac{a(x,y)}{\int \nolimits _F a(x,z)\mu (dz)}. \end{aligned}$$

While considering hermaphroditic populations, one can expect a model with a symmetric mating rate. We obtain such a model assuming that the mating rate is of the form

6 $$\begin{aligned} m(x_i,x_j;{\mathbf {x}})=\frac{a(x_i,x_j)}{2\sum \nolimits _{l=1}^n a(x_i,x_l)}+\frac{a(x_i,x_j)}{2\sum \nolimits _{l=1}^n a(x_j,x_l)}, \end{aligned}$$

where $a(x,y)$ is a symmetric nonnegative preference function, e.g. $a(x,y)=\varphi (\Vert x-y\Vert )$ (in the case of populations without self-fertilization we eliminate the terms with $i=l$ and $j=l$ from the denominators). The mating rate in the infinitesimal model is now of the form

7 $$\begin{aligned} m(x,y;\mu )=\frac{a(x,y)}{2\int \nolimits _F a(x,z)\mu (dz)}+\frac{a(x,y)}{2\int \nolimits _F a(y,z)\mu (dz)}. \end{aligned}$$

In the rest of the paper we will assume that the mating rate $m(x_i, x_j; \mathbf{x })$ is of the form (1) or (6).

### Birth of a new individual

After mating/self-fertilization an offspring is born with probability 1. The trait of the offspring is drawn from a distribution $K(x_i,x_j,dz)$, where $x_i$ and $x_j$ are parental traits. We suppose that for every $x,y\in F$ the measure $K(x,y,\cdot )$ is a Borel probability measure with support contained in the set $F$, and assume that there exist positive constants $c_1,c_2,c_3$ such that

8 $$\begin{aligned} \int \limits _F |z| K(x,y,dz)\le c_1+c_2|x|+c_3|y|, \end{aligned}$$

and

9 $$\begin{aligned} \int \limits _F z K(x,y,dz)=\frac{x+y}{2}. \end{aligned}$$

The above condition has a simple biological interpretation, namely, the expected offspring’s trait is the parental mean trait. Moreover, we suppose that for every $x,y\in F$ and for every Borel set $A\subset F$

10 $$\begin{aligned} K(x,y,A)=K(y,x,A), \end{aligned}$$

and the function

11 $$\begin{aligned} (x,y)\mapsto K(x,y,A) \end{aligned}$$

is measurable.

### Competition and death rates

An individual from the population can die naturally or when competing with others. Let us denote by $I(x_i)$ the rate of interaction of the individual with trait $x_i$. We assume that $I$ is a nonnegative function. For individuals with traits $x_i$ and $x_j$ we define a competition kernel $U(x_i,x_j),$ which is assumed to be a nonnegative and symmetric function. Competition always leads to death of one of the competitors. We assume that the natural death rate of the individual with trait $x_i$ is expressed by a number $D(x_i)$, and suppose that $D$ is a nonnegative function.

## Stochastic process corresponding to the model

### The dynamics of the population

We present the dynamics of the ecological system that we are interested in. The process starts at time $t=0$ from an initial distribution. Individuals with traits $x_i$ and $x_j$ can mate at rate $m(x_i,x_j;{\mathbf {x}})$ of the form (1) or (6). After mating an offspring is born with probability one. An individual with trait $x_i$ dies naturally at rate $D(x_i)$ or by competition at rate $I(x_i)\sum _{j}U(x_i,X_j(t)),$ where the sum extends over all living individuals at time $t$, and $X_j(t)$ are their traits. We assume that all the events (mating, natural death, competition) are independent.

### The phase space

We denote by ${\mathbb {N}}$ the set of all positive integers, $\delta _x$ stands for the Dirac measure concentrated at a point $x,$ and ${\mathbb {1}}_A$ denotes the indicator function of a set $A$. We consider the space $M(F)$ of all finite positive Borel measures on $F$ equipped with the topology of weak convergence of measures. We introduce the set ${\mathcal {M}}\subset M(F)$ of the form

12 $$\begin{aligned} {\mathcal {M}}=\left\{ \sum _{i=1}^n \delta _{x_i}:\, n\in {\mathbb {N}},\, x_i\in F\right\} . \end{aligned}$$

For any measure $\mu \in M(F)$ and any measurable function $f$ we define $\left<\mu , f\right>=\int _F f\, d\mu$. In particular, $\left<\mu , f\right> =\sum _{i=1}^n f(x_i)$ if $\mu = \sum _{i=1}^n \delta _{x_i}$. We write ${\mathcal {D}}([0,\infty ), {\mathcal {M}})$ for the Skorokhod space of all cad-lag functions from the interval $[0,\infty )$ to the set ${\mathcal {M}}$ (see for details, e.g., Ethier and Kurtz 1986, Skorokhod 1956).

### Generator of the process

We consider a continuous time ${\mathcal {M}}$-valued stochastic process $(\nu _t)_{t\ge 0}$ with the infinitesimal generator $L$ given for all bounded and measurable functions $\phi :{\mathcal {M}}\rightarrow {\mathbb {R}}$ by the formula

13 $$\begin{aligned} \begin{aligned} L\phi (\nu )&=\int \limits _F \int \limits _F \int \limits _{F}[\phi (\nu +\delta _{z})-\phi (\nu )]m(x,y;\nu )K(x,y,dz)\nu (dx)\nu (dy)\\&\quad +\int \limits _F [\phi (\nu -\delta _{x})-\phi (\nu )]\left( D(x)+I(x)\int \limits _{F}U(x,y)\nu (dy)\right) \nu (dx). \end{aligned} \end{aligned}$$

The first term in the right-hand side describes the mating and birth processes with the dispersal of traits. The second term stands for two kinds of death. The death part of the generator was previously studied in Fournier and Méléard (2004). Notice that, on the contrary to Fournier and Méléard (2004), the operator $L$ has the first term nonlinear with respect to $\nu$.

We assume that there are positive constants $\underline{a}, \overline{a}, \overline{D}, \overline{I}, \overline{U}$ such that for every $x,y\in ~F$

14 $$\begin{aligned} \underline{a}\le a(x,y)\le \overline{a}, \quad p(x)\le \overline{p}, \quad D(x)\le \overline{D}, \quad I(x)\le \overline{I}, \quad U(x,y)\le \overline{U}. \end{aligned}$$

Under the above assumptions, if the initial measure $\nu _0\in {\mathcal {M}}$ satisfies ${\mathbb {E}}\big (\left<\nu _0 , 1\right>^q\big )<\infty$ for some number $q\ge 1$, then

$$\begin{aligned} {\mathbb {E}}\left( \sup _{0\le t\le T}\left<\nu _t , 1\right>^q\right) <\infty \end{aligned}$$

for any $T<\infty$. Consequently, the standard approach of Fournier and Méléard can be easily applied to prove the existence of the Markov process $(\nu _t)_{t\ge 0}$ with the infinitesimal generator given by formula (13) (see Fournier and Méléard 2004, Remenik (2009) for detailed proofs).

## Macroscopic model

### Macroscopic approximation

This section contains an approximation of the process which was introduced and studied in the previous sections. The idea is to normalize the initial model and pass with the number of individuals to infinity, assuming that the “mass” of each individual becomes negligible. In this approximation mating and death rates remain unchanged. Only the intensity of interaction is rescaled, and tends to 0 with an unbounded growth of the population. This approach leads to a deterministic nonlinear integro-differential equation whose solutions describe an evolution of trait distribution.

We consider a sequence of populations indexed by numbers $N\in {\mathbb {N}}$. If the $N$th population consists of individuals ${\mathbf {x}}^N=\{x_1^N,\ldots , x_{n(N)}^N\}$, then

- individuals with traits $x_i^N$ and $x_j^N$ can mate with rate $m(x_i^N,x_j^N;{\mathbf {x}}^N)$ of the form (1) or (6),
- a new offspring’s trait is drawn from a distribution $K(x_i^N,x_j^N,dz)$, where $x_i^N, x_j^N$ are the traits of parents,
- an individual with trait $x_i^N$ can die with rate $D(x_i^N)$,
- an individual with trait $x_i^N$ interacts with other individuals with intensity $I(x_i^N)/N$,
- a competition kernel of individuals with traits $x_i^N, x_j^N$ is a symmetric, nonnegative function $U(x_i^N,x_j^N)$.

The $N$th population is described by a process $(\nu ^N_t)_{t\ge 0}$ which is defined in the same way as the process $(\nu _t)_{t\ge 0}$ but with the corresponding coefficients. We define the ${\mathcal {M}}^N$-valued Markov process $(\mu _t^N)_{t\ge 0}$ by the formula $\mu _t^N=\nu _t^N/N$. The value space for the process $(\mu ^N_t)_{t\ge 0}$ is thus

$$\begin{aligned} {\mathcal {M}}^N=\bigg \{\frac{1}{N}\nu :\nu \in {\mathcal {M}}\bigg \}. \end{aligned}$$

The generator $L^N$ of the process $(\mu _t^N)_{t\ge 0}$ is given by

$$\begin{aligned} L^N\phi (\nu )&= N\int \limits _F \int \limits _F \int \limits _F\bigg (\phi \Big (\nu +\frac{1}{N}\delta _{z}\Big )-\phi (\nu )\bigg )m(x,y;\nu )K(x,y,dz) \,\nu (dx)\,\nu (dy)\\&+\,\,N\int \limits _F \bigg ( \phi \Big (\nu -\frac{1}{N}\delta _{x}\Big )-\phi (\nu )\bigg )\bigg (D(x)+I(x) \int \limits _{F}U(x,y)\,\nu (dy)\bigg )\,\nu (dx). \end{aligned}$$

for any measurable and bounded map $\phi :{\mathcal {M}}^N\rightarrow {\mathbb {R}}$.

#### **Theorem 1**

We assume that condition (14) holds, and the functions $a, p, k, D, I, U$ are continuous. We suppose that ${\mathbb {E}}\big (\left<\mu _0^N , 1\right>^q\big )<\infty$ for some $q\ge 2$ and all $N\in {\mathbb {N}}$, and almost each sequence $(\mu _0^N)_{N\in {\mathbb {N}}}$ converges weakly to a deterministic finite measure $\mu _0$, as $N\rightarrow \infty$. Then for all $T>0$ the sequence of processes $(\mu _t^N)_{N\in {\mathbb {N}}}$ converges in distribution in ${\mathcal {D}}([0,T],M(F))$ to a deterministic and continuous measure-valued function $[0,T] \ni t\mapsto \mu _t\in M(F)$, satisfying the following equation

15 $$\begin{aligned} \left<\mu _t , f\right>&= \left<\mu _0 , f\right>+\int \limits _0^t\int \limits _F\int \limits _F\int \limits _F f(z)m(x,y;\mu _s) K(x,y,dz)\,\mu _s(dx)\,\mu _s(dy)\,ds \nonumber \\&-\int \limits _0^t\int \limits _F f(x)\Big ( D(x)+I(x)\int \limits _F U(x,y)\,\mu _s(dy) \Big ) \mu _s(dx)\,ds \end{aligned}$$

for every bounded and measurable function $f:F\rightarrow {\mathbb {R}}.$

The standard proof of the above theorem is based on Ethier and Kurtz (1986) Corollary 8.16, Chapter 4, and since mating is described by the Lipschitz continuous operator on the space of positive and finite Borel measures with total variation norm, it can be directly adapted for example, from Rudnicki and Wieczorek (2006a).

### Strong solutions in the space of measures

According to Theorem 1 the solutions of (15) are continuous in the topology of weak convergence of measures. In this part we show a stronger result that they are also continuous in the total variation norm $\Vert \nu \Vert _{TV}=\sup \{ |\left<\nu , f\right>|:\,\,f \mathrm{- measurable,} \sup \limits _{x\in F}|f(x)|\le 1\}$. We use the following formal notation

16 $$\begin{aligned} \frac{d}{d t} \mu _t(dz)&= \int \limits _F\int \limits _F \,m(x,y;\mu _t)\, K(x,y,dz)\mu _t(dx)\mu _t(dy)\nonumber \\&-\bigg (D(z)+I(z)\int \limits _F U(z,y)\mu _t(dy)\bigg )\mu _t(dz), \end{aligned}$$

of equation (15) in the space of positive, finite Borel measures $M(F)$ on the set $F$ with the total variation norm.

#### **Theorem 2**

Assume that the functions $a, p, D, I, U$ and (11) are measurable, and condition (14) holds. Moreover, suppose that there exist positive constants $\underline{p}, \underline{I}, \underline{U}$ such that

17 $$\begin{aligned} p(x)\ge \underline{p}, \quad I(x)\ge \underline{I}, \quad U(x,y)\ge \underline{U}, \end{aligned}$$

for all $x,y\in F$. If $\mu _0\in M(F)$, then there exists a unique solution $\mu _t$, $t\ge 0$, of Eq. (16) with the initial condition $\mu _0$. The function $t\mapsto \mu _t$ is bounded and continuous in the norm $\Vert \cdot \Vert _{TV}$.

#### *Proof*

Let us fix $T\ge 0$, $\delta >0$ and consider the space $C_T=C([T,T+\delta ], M(F))$ with the norm $\Vert \mu _\cdot \Vert _T=\sup _{t\in [T,T+\delta ]} \Vert \mu _t\Vert _{TV}$. Define the operator $\Lambda :C_T\rightarrow C_T$ by the formula

$$\begin{aligned} (\Lambda \mu _\cdot )(t)(dz)&=\mu _T(dz)+\int \limits _T^t \int \limits _F\int \limits _F m(x,y;\mu _s)K(x,y,dz)\,\mu _s(dx)\,\mu _s(dy)\,ds\\&\quad -\int \limits _T^t\bigg (D(z)+I(z)\int \limits _F U(z,y)\mu _s(dy)\bigg )\mu _s(dz)\,ds, \end{aligned}$$

where $\mu _T\in M(F)$ is some measure. Notice that from assumption (14) there are constants $\overline{m}, \hat{m}$ depending on $\overline{p}$ in the case of semi-random mating, and on $\underline{a}, \overline{a}$ in the case of assortative mating, such that for any $y\in F$ and measures $\mu , \nu \in M(F)$

18 $$\begin{aligned} \int \limits _F m(x,y;\nu )\,\nu (dx)\le \overline{m}, \end{aligned}$$

and

19 $$\begin{aligned} \int \limits _F\int \limits _F|m(x,y;\mu )-m(x,y;\nu )|\,\mu (dx)\,\nu (dy)\le \hat{m} \Vert \mu -\nu \Vert _{TV}. \end{aligned}$$

Take functions $\mu _\cdot , \nu _\cdot \in C_T$ from the ball $B(0,2\Vert \mu _T\Vert _{TV})$. Then

20 $$\begin{aligned} \Vert \Lambda \mu _\cdot \Vert _T \le \Vert \mu _T\Vert _{TV}+2\delta (\overline{m}+\overline{D}) \Vert \mu _T\Vert _{TV}+4\delta \overline{I}\overline{U} \Vert \mu _T\Vert _{TV}^2, \end{aligned}$$

and

21 $$\begin{aligned} \Vert \Lambda \mu _\cdot -\Lambda \nu _\cdot \Vert _T\le \delta \big (\overline{D}+ 2\overline{m} + 4\hat{m}\Vert \mu _T\Vert _{TV}^2\big ) \Vert \mu _\cdot -\nu _\cdot \Vert _T + 4\delta \overline{I}\overline{U}\Vert \mu _T\Vert _{TV}\Vert \mu _\cdot -\nu _\cdot \Vert _T. \end{aligned}$$

Taking $\delta >0$ sufficiently small, from (20) and (21) it follows that $\Lambda$ transforms the ball $B(0,2\Vert \mu _T\Vert _{TV})$ into itself, and is Lipschitz continuous with some constant $L<1$. By the Banach fixed point theorem the operator $\Lambda$ has a unique fixed point and, consequently, (16) has a unique local solution.

In order to extend a local solution to the whole interval $[0,\infty )$ it is sufficient to show that the solution is bounded. Notice that

22 $$\begin{aligned} \frac{d}{dt}\Vert \mu _t\Vert _{TV}\le \Vert \mu _t\Vert _{TV} \big (\overline{m} -\underline{I}\,\underline{U} \Vert \mu _t\Vert _{TV}\big ), \end{aligned}$$

and consequently

$$\begin{aligned} \Vert \mu _t\Vert _{TV}\le \max \left\{ \Vert \mu _0\Vert _{TV}, \frac{\overline{m}}{\underline{I}\,\underline{U}}\right\} , \end{aligned}$$

which completes the proof of the global existence and uniqueness.

Eventually, we show that the measure $\mu _t$ is positive for $t>0$. Indeed, for any Borel set $A$ we can write $\frac{d}{dt} \mu _t(A)\ge -\phi (t)\mu _t(A)$, where $\phi (t)=\left( \overline{D}+\overline{I}\overline{U}\mu _t(F)\right) .$ Hence

$$\begin{aligned} \mu _t(A)\ge \mu _0(A) \exp \left\{ -\int \limits _0^t\phi (s)\,ds\right\} \ge 0. \end{aligned}$$

$$\square $$

A straight-forward conclusion is the following statement about solutions in $L^1$ space.

#### **Corollary 1**

Suppose that for each $x,y\in F$ the measure $K(x,y,dz)$ is absolutely continuous with respect to the Lebesgue measure and

23 $$\begin{aligned} K(x,y,dz)=k(x,y,z)\,dz. \end{aligned}$$

Under the assumptions of Theorem 2, if $\mu _0$ has a density $u_0\in L^1$ with respect to the Lebesgue measure, then $\mu _t$ also has a density $u(t,\cdot )\in L^1$ and $u(t,z)$ is the unique solution of the following equation

24 $$\begin{aligned} \frac{\partial }{\partial t} u(t,z)&= \int \limits _F\int \limits _F \,m(x,y;u(t,\xi )d\xi )\, k(x,y,z)u(t,x)u(t,y)\,dx\,dy\nonumber \\&-\left( D(z)+I(z)\int \limits _F U(z,y)u(t,y)\,dy\right) u(t,z), \end{aligned}$$

with the initial condition $u(0,\cdot )=u_0(\cdot )$.

#### *Proof*

Take a Borel set $A$ with zero Lebesgue measure. Since the measure $K(x,y,dz)$ is absolute continuous with respect to the Lebesgue measure, $K(x,y,A)=0$ for every $x,y\in F,$ and consequently $\frac{d}{d t} \mu _t(A)\le -\underline{D} \,\mu _t(A)$, and $\mu _0(A)=0$. Therefore $\mu _t(A)=0$ for all $t>0$, and the statement comes from the Radon-Nikodym theorem. $\square$

### Boundedness, extinction and persistence

From the proof of Theorem 2 it follows that the function $M(t)=\mu _t(F)$ is upper-bounded. Now we analyze further properties of $M(t)$. Let us recall that a population *becomes extinct* if $\lim _{t \rightarrow \infty }M(t)=0$, and *is persistent* provided $\liminf _{t \rightarrow \infty }M(t)>0$.

#### **Proposition 1**

If $\inf _z D(z)\ge \sup _z p(z)$ in the case of random mating and $\inf _z D(z)\ge 1$ in the case of assortative mating, then the population becomes extinct. If $\sup _z D(z)< \inf _z p(z)$ in the case of random mating and $\sup _z D(z)< 1$ in the case of assortative mating, then the population is persistent.

#### *Proof*

In the case of random mating these properties follow simply from the inequalities

$$\begin{aligned} M'(t)\le M(t)\left( \bar{p} - \underline{D} - \underline{I}\,\underline{U} M(t)\right) \end{aligned}$$

and

$$\begin{aligned} M'(t)\ge M(t)\left( \underline{p} - \overline{D} - \overline{I}\,\overline{U} M(t)\right) . \end{aligned}$$

In the case of assortative mating we use similar inequalities with $\bar{p}$ and $\underline{p}$ replaced by 1. $\square$

### Equation on a global attractor

In order to describe more precisely asymptotic behavior of $M(t)$, we need to assume that the functions $p, D, I, U$ do not depend on $x$, and are positive. To avoid extinction of the population, we additionally assume that $D<p$ in the case of random mating and $D<1=:p$ in the case of assortative mating (see Proposition 1). Then $M(t)$ satisfies the following equation

$$\begin{aligned} M'(t)=pM(t)-(D+IUM(t))M(t), \end{aligned}$$

and $\bar{M}=(p-D)/ (IU)$ is a stationary solution of this equation. Using basic facts from the theory of differential equations, it is easy to see that

25 $$\begin{aligned} \lim _{t\rightarrow \infty } M(t)=\bar{M}. \end{aligned}$$

The number $\bar{M}$ is an analogue of *carrying capacity* studied in Bolker and Pacala (1997) and Fournier and Méléard (2004). In our case $\bar{M}$ is a number of individuals per unit of volume after long time.

From (25) it follows that all positive solutions converge to the set

$$\begin{aligned} {\mathcal {A}}=\big \{\mu \in M(F):\,\,\mu (F)=\bar{M}\big \}, \end{aligned}$$

which is invariant with respect to Eq. (16), i.e., if an initial condition $\mu _0$ belongs to ${\mathcal {A}}$, then $\mu _t \in {\mathcal {A}}$ for $t>0$. It means that ${\mathcal {A}}$ is a *global attractor* for Eq. (16). If $\mu _0\in {\mathcal {A}}$ then the function $t\mapsto \mu _t$ satisfies the following equation

26 $$\begin{aligned} \frac{d}{d t} \mu _t(dz)=\int \limits _F\int \limits _F \,m(x,y;\mu _t)\, K(x,y,dz)\,\mu _t(dx)\,\mu _t(dy) -p \mu _t(dz). \end{aligned}$$

Let $\mu _t$ be a positive solution of (16). If we substitute $\bar{\mu }_t=\frac{\bar{M}}{M(t)}\mu _t$, then the function $t\mapsto \bar{\mu }_t$ also satisfies (26). Therefore, the long-time behaviour of solutions of (26) is completely characterized by the dynamics on the attractor ${\mathcal {A}}$.

Now we consider only solutions on the set ${\mathcal {A}}$. If we replace $\mu _t(dx)$ by $\bar{M}\mu _t(dx)$ and $t$ by $p t$ in (26), then $\mu _t(dz)$ becomes a probability measure for all $t\ge 0$. The function $t\mapsto \mu _t$ satisfies

27 $$\begin{aligned} \frac{d}{dt} \mu _t(dz)+ \mu _t(dz)=\int \limits _F\int \limits _F K(x,y,dz)\mu _t(dx)\mu _t(dy), \end{aligned}$$

in the case of random mating, and

28 $$\begin{aligned} \frac{d}{dt} \mu _t(dz)+ \mu _t(dz)=\int \limits _F\int \limits _F \psi (x,y;\mu _t)K(x,y,dz)\mu _t(dx)\mu _t(dy), \end{aligned}$$

in the case of assortative mating, where

$$\begin{aligned} \psi (x,y;\mu )=\frac{a(x,y)}{2\int \nolimits _F a(x,r)\mu (dr)}+\frac{a(x,y)}{2\int \nolimits _F a(y,r)\mu (dr)}. \end{aligned}$$

## Asymptotic stability in the case of random mating

### General remarks

In this section we study the convergence of solutions of Eq. (27) to some stationary solutions. Equation (27) can be treated as an evolution equation

29 $$\begin{aligned} \mu _t'={\mathcal {P}}\mu _t-\mu _t , \end{aligned}$$

where the operator ${\mathcal {P}}$ acting on the space of all probability Borel measures on $F$ is given by the formula

30 $$\begin{aligned} ({\mathcal {P}}\mu )(A)=\int \limits _F\int \limits _F K(x,y,A)\,\mu (dx)\,\mu (dy). \end{aligned}$$

The solution of (29) with an initial measure $\mu _0$ is the deterministic process $\mu _t$ given by Theorem 2. The set ${\mathcal {O}}(\mu _0):=\{\mu _t:t\ge 0\}$ is called the *orbit* of $\mu _0$.

Since the problem of the asymptotic stability of the solutions of Eq. (29) in an arbitrary $d$-dimensional space seems to be quite difficult, we consider only the case when $d=1$ and $F$ is a closed interval with nonempty interior. Generally, Eq. (29) has a lot of different stationary measures and it is rather difficult to predict a limit of a given solution. Assumption (9) allows us to omit this difficulty. Indeed, if a measure $\mu$ has a finite first moment $q$, then according to (8) and (9) we have

$$\begin{aligned} \int \limits _F |z|\,(\mathcal {P}\mu )(dz)&= \int \limits _F\int \limits _F\int \limits _F |z|K(x,y,dz)\,\mu (dx)\,\mu (dy)\\&\le \int \limits _F\int \limits _F (c_1+c_2|x|+c_3|y|)\, \mu (dx)\,\mu (dy)\\&\le c_1+(c_2+c_3)\int \limits _F|x|\,\mu (dx)<\infty \end{aligned}$$

and

$$\begin{aligned} \int \limits _F z\,(\mathcal {P}\mu )(dz)\!=\!\int \limits _F\int \limits _F\int \limits _F zK(x,y,dz)\,\mu (dx)\,\mu (dy)\!=\!\int \limits _F\int \limits _F \frac{x+y}{2}\, \mu (dx)\,\mu (dy)\!=\!q. \end{aligned}$$

Therefore, any solution $\mu _t$ of Eq. (29) has the same first moment for all $t\ge 0$. It means that we can restrict our consideration only to probability Borel measures with the same first moment.

The following example shows why we consider solutions of Eq. (29) with values in the space of probability Borel measures instead of the space of probability densities. In this example all the stationary solutions are the Dirac measures, and any solution converges in the weak sense to some stationary measure.

#### *Example 1*

Let $Z$ be a random variable with values in the interval $[-1,1]$ such that ${\mathbb {E}}Z=0$ and $|Z|\not \equiv 1.$ Assume that if $x$ and $y$ are parental traits, then the trait of an offspring is given by

$$\begin{aligned} \frac{x+y}{2}+ Z\frac{|x-y|}{2}, \end{aligned}$$

i.e., the trait of an offspring is distributed between the traits of parents according to the law of $Z$. For a random variable $X$ we denote by $m_1(X)$ and $m_2(X)$ its first and second moments and by $D(X)$ its variance, i.e., $D(X)=m_2(X)-(m_1(X))^2$. Let $\mu _t$, $t\ge 0$, be a solution of (29) with a finite second moment, and let $X_t$, $t\ge 0$, be random variables with distribution measures $\mu _t$. Then $\bar{x}:=m_1(X_t)$ is a constant, and

31 $$\begin{aligned} \frac{d}{dt} D(X_t)= -\frac{1}{2} (1-D(Z))D(X_t). \end{aligned}$$

Since $D(Z)<1$, we have $\lim _{t\rightarrow \infty } D(X_t)=0$. Consequently, $\mu _t$ converges weakly to $\delta _{\bar{x}}$.

### The Wasserstein distance

In order to investigate asymptotic properties of the solutions, we recall some basic facts concerning the Wasserstein distance between measures. For $\alpha \ge 1$ we denote by ${\mathcal {M}}_{\alpha }$ the set of all probability Borel measures $\mu$ on $F$ such that $\int _F |z|^{\alpha }\,\mu (dz)<\infty$ and by ${\mathcal {M}}_{\alpha ,q}$ the subset of ${\mathcal {M}}_{\alpha }$ which contains all the measures such that $\int _F z\,\mu (dz)=q$. For any two measures $\mu ,\nu \in {\mathcal {M}}_1$, we define the *Wasserstein distance* by the formula

32 $$\begin{aligned} d(\mu ,\nu )=\sup _{f\in \mathrm{Lip}_1} \int \limits _F f(z)(\mu -\nu )(dz), \end{aligned}$$

where $\hbox {Lip}_1$ is the set of all continuous functions $f:F\rightarrow {\mathbb {R}}$ such that for any $x,y\in F$

$$\begin{aligned} |f(x)-f(y)|\le |x-y|. \end{aligned}$$

The following lemma is of a great importance in the subsequent part of the paper.

#### **Lemma 1**

The Wasserstein distance between measures $\mu ,\nu \in {\mathcal {M}}_{1}$ can be computed by the formula

33 $$\begin{aligned} d(\mu ,\nu )=\int \limits _F |\Phi (x)|\,dx, \end{aligned}$$

where $\Phi (z)=(\mu -\nu )\left( F\cap (-\infty ,z]\right)$ is the cumulative distribution function of the signed measure $\mu -\nu$.

#### *Proof*

Let $\Phi _{\mu }$ and $\Phi _{\nu }$ be the cumulative distribution functions of the measures $\mu$ and $\nu$. Since these measures have finite first absolute moments we have

$$\begin{aligned} \lim _{x\rightarrow -\infty }|x|\Phi _{\mu }(x) =\lim _{x\rightarrow -\infty }|x|\Phi _{\nu }(x)=0 \end{aligned}$$

and

$$\begin{aligned} \lim _{x\rightarrow \infty }x(1-\Phi _{\mu }(x)) =\lim _{x\rightarrow \infty }x(1 -\Phi _{\nu }(x))=0. \end{aligned}$$

This gives

$$\begin{aligned} \limsup _{x\rightarrow \pm \infty }|f(x)|\Phi (x) \le \lim _{x\rightarrow \pm \infty }|x|\Phi (x) =0\quad \mathrm{for}\,f\in \hbox {Lip}_1 \end{aligned}$$

and if $F$ is bounded from below or from above, then we have $\Phi (x)=0$ for $x\notin F$. Since $f$ is a locally absolutely continuous function, integrating by parts leads to the formula

$$\begin{aligned} d(\mu ,\nu )=\sup _{f\in \mathrm{Lip}_1} \int \limits _F f(z)\,d\Phi (z)=\sup _{f\in \mathrm{Lip}_1} -\int \limits _F f'(z)\Phi (z)\,dz. \end{aligned}$$

Clearly the supremum is taken when $f'(z)=-\mathrm{sgn }\Phi (z)$. $\square$

Consider probability measures $\mu$ and $\mu _n$, $n\in {\mathbb {N}}$, on the set $F$. We recall that the sequence $(\mu _n)$ converges *weakly* (or *in a weak sens*) to $\mu$, if for any continuous and bounded function $f:F\rightarrow {\mathbb {R}}$

$$\begin{aligned} \int \limits _F f(x)\,\mu _n(dx)\rightarrow \int \limits _F f(x)\,\mu (dx), \end{aligned}$$

as $n\rightarrow \infty$. It is well-known that the convergence in the Wasserstein distance implies the weak convergence of measures. Moreover, the space of probability Borel measures on any complete metric space is also a complete metric space with the Wasserstein distance (see e.g. Bolley 1934; Rachev 1991). The convergence of the sequence $\mu _n$ to $\mu$ in the space ${\mathcal {M}}_{1,q}$ is equivalent to the following condition (see Villani (2008), Definition 6.7 and Theorem 6.8)

C $$\begin{aligned} \mu _n\rightarrow \mu \text{ weakly, } \text{ as } n\rightarrow \infty \quad \text{ and } \quad \lim _{R\rightarrow \infty } \limsup _{n\rightarrow \infty } \int \limits _{F_R} |x|\mu _n(dx)=0, \end{aligned}$$

where $F_R:=\{x\in F:|x|\ge R \}.$ Fix $q\in F$, $\alpha >1$, and $m>0$. Consider a set $\widetilde{{\mathcal {M}}} \subset {\mathcal {M}} _{1,q}$ such that

34 $$\begin{aligned} \int \limits _F |x|^\alpha \mu (dx)\le m \end{aligned}$$

for all $\mu \in \widetilde{{\mathcal {M}}}.$ Then the set $\widetilde{{\mathcal {M}}}$ is relatively compact in ${\mathcal {M}}_{1,q}$. Indeed, by Markov inequality, $\mu (\{x:|x|\le R\})\ge 1-m/R^\alpha$ for all $\mu \in \widetilde{{\mathcal {M}}}$, what means that the set $\widetilde{{\mathcal {M}}}$ is tight, and thus $\widetilde{{\mathcal {M}}}$ is relatively compact in the topology of weak convergence (see e.g. Billingsley (1995)). Moreover, for $\mu \in \widetilde{{\mathcal {M}}}$ we have

$$\begin{aligned} \int \limits _{F_R} |x|\mu _k(dx)\le \frac{1}{R^{\alpha -1}} \int \limits _{F_R} |x|^\alpha \mu _k(dx)\le \frac{m}{R^{\alpha -1}}, \end{aligned}$$

which implies the second condition in (C). Consequently, the set $\widetilde{{\mathcal {M}}}$ is relatively compact in ${\mathcal {M}}_{1,q}$.

### Theorems on asymptotic stability

We use the script letter ${\mathcal {K}}$ for the cumulative distribution function of the measure $K$, i.e.,

$$\begin{aligned} {\mathcal {K}}(x,y,z)= K(x,y,F\cap (-\infty ,z]). \end{aligned}$$

The main result of this section is the following.

#### **Theorem 3**

Fix $q\in F$. Suppose that

(i) for all $y,z\in F$ the function ${\mathcal {K}}(x,y,z)$ is absolutely continuous with respect to $x$ and for each $a,b,y\in F$ we have
  35 $$\begin{aligned} \int \limits _F \Big |\frac{\partial }{\partial x}{\mathcal {K}}(a,y,z)-\frac{\partial }{\partial x}{\mathcal {K}}(b,y,z)\Big |\,dz< 1, \end{aligned}$$
(ii) there are constants $\alpha >1$, $L<1$, and $C\ge 0$ such that for every $\mu \in {\mathcal {M}}_{\alpha ,q}$ we have
  36 $$\begin{aligned} \int \limits _F |x|^{\alpha }\mathcal {P}\mu (dx)\le C+L\int \limits _F |x|^{\alpha } \mu (dx). \end{aligned}$$
  Then for every initial measure $\mu _0\in {\mathcal {M}}_{1,q}$ there exists a unique solution $\mu _t$, $t\ge 0$, of Eq. (27) with values in ${\mathcal {M}}_{1,q}$. Moreover, there exists a unique measure $\mu ^*\in {\mathcal {M}}_{1,q}$ such that ${\mathcal {P}}\mu ^*=\mu ^*$ and for every initial measure $\mu _0\in {\mathcal {M}}_{1,q}$ the solution $\mu _t$, $t\ge 0$, of Eq. (27) converges to $\mu ^*$ in the space ${\mathcal {M}}_{1,q}$.

We split the proof of Theorem 3 into a sequence of lemmas. Denote by ${\mathcal {F}}_q$ the set of all cumulative distribution functions of the signed measures of the form $\mu -\nu$, where $\mu ,\nu \in {\mathcal {M}}_{1,q}$.

#### **Lemma 2**

Suppose that for all $y\in F$ and $\Phi \in {\mathcal {F}}_q$, $\Phi \not \equiv 0$, we have

37 $$\begin{aligned} 2\int \limits _F\left| \int \limits _F {\mathcal {K}}(x,y,z)\Phi (dx)\right| \,dz <\int \limits _F |\Phi (x)|\,dx. \end{aligned}$$

Then

38 $$\begin{aligned} d(\mathcal {P}\mu ,\mathcal {P}\nu )<d(\mu ,\nu ) \end{aligned}$$

for $\mu ,\nu \in {\mathcal {M}}_{1,q}$, $\mu \ne \nu$. In particular, for every initial measure $\mu _0\in {\mathcal {M}}_{1,q}$ there exists a unique solution $\mu _t$, $t\ge 0$, of Eq. (27) with values in ${\mathcal {M}}_{1,q}$. $\square$

#### *Proof*

Since $K(x,y,\cdot )=K(y,x,\cdot )$, we can write

$$\begin{aligned} \mathcal {P}\mu -\mathcal {P}\nu = 2\int \limits _F\int \limits _F K(x,y,\cdot )\,(\mu -\nu )(dx)\,\bar{\mu }(dy), \end{aligned}$$

where $\bar{\mu }=(\mu +\nu )/2$. If $\Phi (x)$ is the cumulative distribution function of $\mu -\nu$, then the signed measure $\mathcal {P}\mu -\mathcal {P}\nu$ has the cumulative distribution function of the form

$$\begin{aligned} 2\int \limits _F\int \limits _F {\mathcal {K}}(x,y,z)\,\Phi (dx)\,\bar{\mu }(dy). \end{aligned}$$

Hence

$$\begin{aligned} d(\mathcal {P}\mu ,\mathcal {P}\nu )&=2\int \limits _F \left| \int \limits _F\int \limits _F {\mathcal {K}}(x,y,z)\,\Phi (dx)\,\bar{\mu }(dy)\right| dz\\&\le 2\int \limits _F \int \limits _F\left| \int \limits _F {\mathcal {K}}(x,y,z)\,\Phi (dx)\right| \,dz\, \bar{\mu }(dy)\\&<\int \limits _F \int \limits _F\left| \Phi (x)\right| \,dx\,\bar{\mu }(dy) =\int \limits _F\left| \Phi (x)\right| \,dx=d(\mu ,\nu ) . \end{aligned}$$

$$\square $$

#### **Lemma 3**

Suppose that condition (i) of Theorem 3 is fulfilled. Then condition (37) holds.

#### *Proof*

Take a $\Phi \in {\mathcal {F}}_q$ and denote $\Phi ^+(x)=\max \{0,\Phi (x)\}$ and $\Phi ^-(x)=\max \{0,-\Phi (x)\}$. Since $\Phi$ is the cumulative distribution function of $\mu -\nu$, where $\mu ,\nu \in {\mathcal {M}}_{1,q}$, we have

$$\begin{aligned} \int \limits _F\Phi (x)\,dx=-\int \limits _F x\,\Phi (dx)=\int \limits _F x\,\Phi _{\nu }(dx)-\int \limits _F x\,\Phi _{\mu }(dx) =0, \end{aligned}$$

and, consequently,

39 $$\begin{aligned} \int \limits _F\Phi ^+(x)\,dx=\int _F\Phi ^-(x)\,dx=\frac{1}{2}\int \limits _F|\Phi (x)|\,dx. \end{aligned}$$

Since $\Phi ^+$ and $\Phi ^-$ are nonnegative functions and have the same integral, condition (35) implies

40 $$\begin{aligned} \int \limits _F \Big |\int \limits _F\frac{\partial }{\partial x}{\mathcal {K}}(x,y,z)\Phi ^+(x)\,dx -\int \limits _F\frac{\partial }{\partial x}{\mathcal {K}}(x,y,z)\Phi ^-(x)\,dx\Big |\,dz< \int \limits _F \Phi ^+(x)\,dx. \end{aligned}$$

Integrating $\int _F {\mathcal {K}}(x,y,z)\Phi (dx)$ by parts we obtain

41 $$\begin{aligned} \int \limits _F {\mathcal {K}}(x,y,z)\Phi (dx)=-\int \limits _F \frac{\partial }{\partial x}{\mathcal {K}}(x,y,z)\Phi (x)\,dx . \end{aligned}$$

From (40) and (41) it follows

$$\begin{aligned} 2\int \limits _F\left| \int \limits _F {\mathcal {K}}(x,y,z)\Phi (dx)\right| \,dz <2\int \limits _F \Phi ^+(x)\,dx=\int \limits _F |\Phi (x)|\,dx. \end{aligned}$$

$$\square $$

#### **Lemma 4**

Assume that $d(\mathcal {P}\mu ,\mathcal {P}\nu )<d(\mu ,\nu )$ for all $\mu ,\nu \in {\mathcal {M}}_{1,q}$, $\mu \ne \nu$. Let $\mu _0,\nu _0\in {\mathcal {M}}_{1,q}$ and denote by $\mu _t$ and $\nu _t$, respectively, the solutions of Eq. (29) in the space of probability Borel measures on $F$. Then $\mu _t,\nu _t\in {\mathcal {M}}_{1,q}$ for $t\ge 0$ and $d(\mu _t,\nu _t)<d(\mu _r,\nu _r)$ for $0\le r<t\le T$ provided that $\mu _T\ne \nu _T$.

#### *Proof*

Since $\mathcal {P}({\mathcal {M}}_{1,q})\subset {\mathcal {M}}_{1,q}$ every solution of (29) with an initial value from the set ${\mathcal {M}}_{1,q}$ remains in this set for all $t\ge 0$. Any solution $\mu _t$ of (29) satisfies the following integral equation

42 $$\begin{aligned} \mu _t=e^{r-t}\mu _r+\int \limits _r^t e^{s-t}\mathcal {P}\mu _s\,ds. \end{aligned}$$

Let $\mu _t$ and $\nu _t$ be solutions of (29) with values in ${\mathcal {M}}_{1,q}$ and such that $\mu _T\ne \nu _T$. Then $\mu _t\ne \nu _t$ for $t\le T$ and from (42) it follows that

$$\begin{aligned} d(\mu _t,\nu _t)&\le e^{r-t}d(\mu _r,\nu _r)+\int \limits _r^t e^{s-t}d(\mathcal {P}\mu _s,\mathcal {P}\nu _s)\,ds\\&<e^{r-t}d(\mu _r,\nu _r)+\int \limits _r^t e^{s-t}d(\mu _s,\nu _s)\,ds \end{aligned}$$

for $0\le r<t\le T$. Let $\alpha (s)=e^sd(\mu _s,\nu _s)$. Then

$$\begin{aligned} \alpha (t)<\alpha (r)+\int \limits _r^t\alpha (s)\,ds \end{aligned}$$

and from Gronwall’s lemma it follows that $\alpha (t)<\alpha (r)e^{t-r}$, which gives $d(\mu _t,\nu _t)<d(\mu _r,\nu _r)$. $\square$

#### **Lemma 5**

Assume that condition (ii) of Theorem 3 is fulfilled. Then for every initial measure $\mu _0\in {\mathcal {M}}_{\alpha ,q}$ its orbit ${\mathcal {O}}(\mu _0)$ is a relatively compact subset of ${\mathcal {M}}_{1,q}$. Moreover, $\mathrm{cl\,}{\mathcal {O}}(\mu _0)\subset {\mathcal {M}}_{\alpha ,q}$, where $\mathrm{cl\,}{\mathcal {O}}(\mu _0)$ denotes the closure of ${\mathcal {O}}(\mu _0)$ in $({\mathcal {M}}_{1,q},d)$.

#### *Proof*

Take a $\mu _0\in {\mathcal {M}}_{\alpha ,q}$ and let $\mu _t$ be a solution of (29) with the initial condition $\mu _0$. Let $m_0=C/(1-L)$, $m_{\alpha }=\int _F |x|^{\alpha }\, \mu _0(dx)$, and $m=\max \{m_0,m_{\alpha }\}$. We check that $\mu _t\in {\mathcal {M}}_{\alpha ,q}$ for $t\ge 0$ and

43 $$\begin{aligned} \int \limits _F |x|^{\alpha }\, \mu _t(dx)\le m \,\mathrm{for}\,t\ge 0. \end{aligned}$$

To see this, we define the set

$$\begin{aligned} {\mathcal {M}}_{\alpha ,q,m}=\left\{ \mu \in {\mathcal {M}}_{1,q}:\int \limits _F |x|^{\alpha }\,\mu (dx)\le m \right\} . \end{aligned}$$

Then ${\mathcal {M}}_{\alpha ,q,m}$ is a closed and convex subset of ${\mathcal {M}}_{1,q}$. Hence $C([0,T], {\mathcal {M}}_{\alpha ,q,m})$ is a closed subset of $C([0,T], {\mathcal {M}}_{1,q})$. For a sufficiently small $T>0$, the map

$$\begin{aligned} (\Lambda \mu )_t=e^{-t}\mu _0+\int \limits _0^t e^{s-t}\mathcal {P}\mu _s\,ds \end{aligned}$$

is a contraction on the space $C([0,T], {\mathcal {M}}_{1,q})$ and the function $t\mapsto \mu _t$, $0\le t\le T$, is its fixed point. In order to prove that $\mu _t\in {\mathcal {M}}_{\alpha ,q}$ for $t\ge 0$ and that (43) holds, it is sufficient to check that the set $C([0,T], {\mathcal {M}}_{\alpha ,q,m})$ is invariant with respect to $\Lambda$. Indeed, since $C+Lm\le m$ we have

$$\begin{aligned} \int \limits _F |x|^{\alpha }\,(\Lambda \mu )_t(dx)&=e^{-t}\int \limits _F |x|^{\alpha }\,\mu _0(dx) +\int \limits _0^t e^{s-t}\int \limits _F |x|^{\alpha }\,\mathcal {P}\mu _s(dx)\,ds \\&\le e^{-t}\int \limits _F |x|^{\alpha }\,\mu _0(dx) +\int \limits _0^t e^{s-t}\bigg ( C+ L\int \limits _F |x|^{\alpha }\,\mu _s(dx)\bigg ) \,ds \\&\le e^{-t}m +\int \limits _0^t e^{s-t}( C+ L m) \,ds\le m. \end{aligned}$$

Thus, there exists $m>0$ depending on $\mu _0$ and $\alpha >1$ such that (43) holds. Consequently, the orbit is a relatively compact subset of ${\mathcal {M}}_{1,q}$ and $\mathrm{cl\,}{\mathcal {O}}(\mu _0)\subset {\mathcal {M}}_{\alpha ,q}$. $\square$

Let $\{S(t)\}_{t\ge 0}$ be a family of transformations of ${\mathcal {M}}_{1,q}$ defined by $S(t)\mu _0=\mu _t$, where $\mu _t$ is the solution of (29) with the initial condition $\mu _0$. For $\mu \in {\mathcal {M}}_{1,q}$ we define the $\omega$-*limit set* by

$$\begin{aligned} \omega (\mu )=\big \{\nu :\nu =\lim _{n\rightarrow \infty }\mu _{t_n}\,\mathrm{for~a~sequence }\, (t_n)_{n\in {\mathbb {N}}}\hbox { with }\lim _{n\rightarrow \infty }t_n=\infty \big \}. \end{aligned}$$

#### *Proof of Theorem 3*

Take a measure $\mu \in {\mathcal {M}}_{\alpha ,q}$. According to Lemma 5 the orbit of $\mu$ is a relatively compact subset of ${\mathcal {M}}_{1,q}$. From this it follows that $\omega (\mu )$ is a nonempty compact set and for $t>0$ we have $S(t)(\omega (\mu ))=\omega (\mu )$. First we check that $\omega (\mu )$ is a singleton. Indeed, if $\omega (\mu )$ has more than one element, then since $\omega (\mu )$ is a compact set, we can find two elements $\nu _1$ and $\nu _2$ in $\omega (\mu )$ with maximum distance $d(\nu _1,\nu _2)$. But since $S(t)(\omega (\mu ))=\omega (\mu )$, then for given $t>0$ there exist $\bar{\nu }_1$ and $\bar{\nu }_2$ in $\omega (\mu )$ such that $S(t)\bar{\nu }_1=\nu _1$ and $S(t)\bar{\nu }_2=\nu _2$. Now from condition (i) and Lemmas 2, 3, and 4 it follows that

$$\begin{aligned} d(\nu _1,\nu _2)= d(S(t)\bar{\nu }_1,S(t)\bar{\nu }_2)< d(\bar{\nu }_1,\bar{\nu }_2), \end{aligned}$$

which contradicts the definition of $\nu _1$ and $\nu _2$. Let $\omega (\mu )=\{\mu ^*\}$. Then $S(t)\mu ^*=\mu ^*$ for $t\ge 0$ and, consequently, ${\mathcal {P}}\mu ^*=\mu ^*$. Since the orbit ${\mathcal {O}}(\mu )$ is relatively compact, we have $\lim _{t\rightarrow \infty } S(t)\mu =\mu ^*$. According to Lemmas 2 and 3 the operator ${\mathcal {P}}$ has only one fixed point what means that the limit $\lim _{t\rightarrow \infty } S(t)\mu$ does not depend on $\mu \in {\mathcal {M}}_{\alpha ,q}$. Now, we consider a measure $\mu \in {\mathcal {M}}_{1,q}$. The set ${\mathcal {M}}_{\alpha ,q}$ is dense in the space ${\mathcal {M}}_{1,q}$. Thus, for every $\varepsilon >0$ we can find $\bar{\mu }\in {\mathcal {M}}_{\alpha ,q}$ such that $d(\mu ,\bar{\mu })< \varepsilon$. Moreover, since $\lim _{t\rightarrow \infty } S(t)\bar{\mu }=\mu ^*$ we find $t_{\varepsilon }$ such that $d(S(t)\bar{\mu },\mu ^*)< \varepsilon$ for $t\ge t_{\varepsilon }$. As the operators $S(t)$ are contractions we have

$$\begin{aligned} d(S(t)\mu ,\mu ^*)\le d(S(t)\mu ,S(t)\bar{\mu })+d(S(t)\bar{\mu },\mu ^*)<2\varepsilon \end{aligned}$$

for $t\ge t_{\varepsilon }$, which completes the proof. $\square$

We can strengthen the thesis of Theorem 3, if we additionally assume that for all $x,y\in F$ the measure $K(x,y,dz)$ has a density $k(x,y,z)$ and $k$ is a bounded and continuous function.

#### **Theorem 4**

Assume that $k$ is a bounded and continuous function, and $k$ satisfies assumptions of Theorem 3. Then the stationary measure $\mu ^*$ is absolutely continuous with respect to the Lebesgue measure and has a continuous and bounded density $u_*(x)$. Moreover, for every $\mu _0\in {\mathcal {M}}_{1,q}$ the solution $\mu _t$ of Eq. (27) can be written in the form $\mu _t=e^{-t}\mu _0+\nu _t$, where $\nu _t$ are absolutely continuous measures, have continuous and bounded densities $v_t(x)$, which converge uniformly to $u_*(x)$.

#### *Proof*

Since $k$ is a continuous and bounded function and $\mu ^*$ is a probability measure,

$$\begin{aligned} u_*(z):=\int \limits _{F}\int \limits _{F}k(x,y,z)\, \mu ^*(dx)\,\mu ^*(dy) \end{aligned}$$

is a continuous bounded function and $u_*$ is a density of $\mu ^*$ because $\mu ^*$ is a fixed point of the operator $\mathcal {P}$. For any initial measure $\mu _0\in {\mathcal {M}}_{1,q}$ the solution $\mu _t$ of (27) satisfies the equation

$$\begin{aligned} \mu _t=e^{-t}\mu _0+\int \limits _0^t e^{s-t}\mathcal {P}\mu _s\, ds. \end{aligned}$$

For each $s\ge 0$ the measure $\mathcal {P}\mu _s$ has a continuous and bounded density $\bar{u}_s(x)$. Since the function $\varphi :[0,\infty )\rightarrow {\mathcal {M}}_{1,q}$ given by $\varphi (s)=\mu _s$ is continuous and $\lim _{s\rightarrow \infty }\mu _s=\mu ^*$, the function $\psi :[0,\infty )\rightarrow C_b(F)$ given by $\psi (s)=\bar{u}_s$ is continuous and $\lim _{s\rightarrow \infty }\bar{u}_s=u_*$. Thus the measures $\nu _t=\int _0^t e^{s-t}\mathcal {P}\mu _s\, ds$ have continuous and bounded densities $v_t(x)$ and $v_t$ converges uniformly to $u_*$ as $t\rightarrow \infty$. $\square$

### Examples

Now, we study two biologically reasonable forms of $K$, which satisfy conditions (i) and (ii) of Theorem 3.

#### *Example 2*

We suppose that if $x$ and $y$ are parental traits, then the trait of the offspring is of the form

$$\begin{aligned} \frac{x+y}{2}+Z, \end{aligned}$$

where $Z$ is a 0-mean random variable, ${\mathbb {E}}Z^2<\infty$ and $Z$ has a positive density $h$. Then

44 $$\begin{aligned} k(x,y,z)=h\left( z-\tfrac{x+y}{2}\right) \end{aligned}$$

and

$$\begin{aligned} \frac{\partial }{\partial x}{\mathcal {K}}(x,y,z)=-\frac{1}{2} h\left( z-\tfrac{x+y}{2}\right) . \end{aligned}$$

The condition (i) is equivalent to the inequality

$$\begin{aligned} \int \limits _{-\infty }^{\infty }|h(z-\alpha )-h(z-\beta )|\,dz<2 \end{aligned}$$

for all $\alpha ,\beta \in {\mathbb {R}}$, which is a simple consequence of the assumption that $h$ is a positive density. Now we check that condition (ii) holds with $\alpha =2$. We have

$$\begin{aligned} \int \limits _{-\infty }^{\infty } z^2(\mathcal {P}\mu )(dz)&\le \int \limits _{-\infty }^{\infty }\int \limits _{-\infty }^{\infty }\int \limits _{-\infty }^{\infty } \Big ((z-\tfrac{x+y}{2})^2 +(z-\tfrac{x+y}{2})(x+y) +\tfrac{(x+y)^2}{4}\Big ) \\&\quad \times h\left( z-\tfrac{x+y}{2}\right) dz\,\mu (dx)\mu (dy)\\&\le {\mathbb {E}}Z^2+\int \limits _{-\infty }^{\infty }\int \limits _{-\infty }^{\infty } \tfrac{(x+y)^2}{4}\mu (dx)\mu (dy)\\&\le {\mathbb {E}}Z^2+\frac{1}{2} q^2+\frac{1}{2} \int \limits _{-\infty }^{\infty } x^2\,\mu (dx). \end{aligned}$$

If we additionally assume that the density $h$ is a continuous function, then according to Theorem 4 the limit measure $\mu ^*$ has a continuous and bounded density $u_*$, $\mu _t=e^{-t}\mu _0+v_t(x)\,dx$, and $v_t$ converges uniformly to $u_*$.

Now we determine the limiting distribution $\mu ^*$. Densities of the measures $\mu ^*$ and $\mu _0$ have the same first moment $q$ and $u_*$ satisfies the equation

45 $$\begin{aligned} u_*(z)=\int \limits _{\mathbb {R}}\int \limits _{\mathbb {R}}h\Big (z-\frac{x+y}{2}\Big )\,u_*(x)\,u_*(y)\,dx\,dy. \end{aligned}$$

Observe that if a probability density $f$ satisfies (45) and $\int _{\mathbb {R}}xf(x)\,dx =0$, then $f(x-q)$ also satisfies (45) and has the first moment $q$. Since $u_*$ is a unique solution of (45) with the first moment $q$ we have $u_*(x)=f(x-q)$ for $x\in {\mathbb {R}}$. Now we construct the density $f$. Consider an infinite sequence of i.i.d. random variables

$$\begin{aligned} Z_{01},Z_{11},Z_{12},Z_{21},\dots ,Z_{24},Z_{31},\dots ,Z_{38},\dots \end{aligned}$$

with density $h$ and define the random variable

$$\begin{aligned} Y=Z_{01}+\frac{Z_{11}+Z_{12}}{2}+\frac{Z_{21}+\dots +Z_{24}}{4}+\frac{Z_{31}+\dots +Z_{38}}{8}+\dots \,\,. \end{aligned}$$

Then ${\mathbb {E}}Y=0$. If $Y_1$ and $Y_2$ are two independent copies of $Y$ and $Z$ is a random variable with density $h$ independent of $Y_1$ and $Y_2$, then

46 $$\begin{aligned} Y\overset{d}{=}Z+\frac{Y_1+Y_2}{2}. \end{aligned}$$

It means that the density $f$ of $Y$ satisfies (45). Let $h_n(x)=2^nh(2^nx)$ for $n\ge 0$ and $x\in {\mathbb {R}}$. From the definition of the random variable $Y$ it follows that

$$\begin{aligned} f=h_0*h_1^{*2}*h_2^{*4}*h_3^{*8}*\dots , \end{aligned}$$

where $f*g$ denotes the convolution of $f$ and $g$.

From (46) it follows immediately that ${\mathbb {E}}Y^2=2{\mathbb {E}}Z^2$. For instance, if $Z$ has a normal distribution with zero mean and standard deviation $\sigma$, then $Y$ has also a normal distribution with zero mean and standard deviation $\sqrt{2}\sigma$.

#### *Example 3*

As in Example 2 we suppose that if $x$ and $y$ are parental traits, then the trait of the offspring is of the form

$$\begin{aligned} (x+y)Z, \end{aligned}$$

where $Z$ is a random variable with values in the interval $[0,1]$, and has a density $h$ such that

47 $$\begin{aligned} \int \limits _0^{\infty } xh(x)\,dx=\frac{1}{2}. \end{aligned}$$

Then $F=[0,\infty )$ and the function $k$ is of the form

48 $$\begin{aligned} k(x,y,z)=\frac{1}{x+y}h\left( \frac{z}{x+y}\right) \end{aligned}$$

for $z\in [0,x+y]$ and $k(x,y,z)=0$ otherwise. Equation (29) with kernel $k$ given by (48) is known as the *general version of Tjon–Wu equation*. If $h={\mathbb {1}}_{[0,1]}$, then this equation is the Tjon–Wu version of the Boltzmann equation (see Bobylev 1976; Krook and Wu 1977; Tjon and Wu 1979). The asymptotic stability of the classical Tjon–Wu equation in $L^1$ space was proven by Kiełek (see Kiełek 1990). Lasota and Traple (see Lasota 2002; Lasota and Traple 1999) proved stability in the general case but in the sense of the weak convergence of measures. If we assume additionally that the support of $h$ contains an interval $(0,\varepsilon )$, $\varepsilon >0$, then this result follows immediately from Theorem 3. Indeed, in that case one can easily compute

$$\begin{aligned} \frac{\partial }{\partial x}{\mathcal {K}}(x,y,z)=-h\left( \frac{z}{x+y}\right) \frac{z}{(x+y)^2}. \end{aligned}$$

Now, condition (35) is equivalent to the inequality

49 $$\begin{aligned} \int \limits _{0}^{\infty }\big |h(\tfrac{z}{\alpha })\tfrac{z}{\alpha ^2}-h(\tfrac{z}{\beta })\tfrac{z}{\beta ^2}\big |\,dz<1 \end{aligned}$$

for all $\alpha ,\beta >0$. This inequality is a simple consequence of positivity of $h$ on the interval $(0,\varepsilon )$ and of the following condition

$$\begin{aligned} \int \limits _{0}^{\infty }h(\tfrac{z}{\alpha })\tfrac{z}{\alpha ^2}\,dz=\int \limits _{0}^{\infty }h(x)x\,dx=\tfrac{1}{2}. \end{aligned}$$

Now we check that the condition (ii) holds with $\alpha =2$. We have

$$\begin{aligned} \int \limits _{0}^{\infty } z^2(\mathcal {P}\mu )(dz)&\le \int \limits _{0}^{\infty }\int \limits _{0}^{\infty }\int \limits _{0}^{\infty } \frac{z^2}{x+y}h\left( \frac{z}{x+y}\right) dz\,\mu (dx)\mu (dy)\\&\le {\mathbb {E}}Z^2 \int \limits _{0}^{\infty }\int \limits _{0}^{\infty } (x+y)^2 \,\mu (dx)\mu (dy)\\&\le {\mathbb {E}}Z^2\Big (2q^2+2 \int \limits _{0}^{\infty } y^2 \,\mu (dy)\Big )\le 2q^2{\mathbb {E}}Z^2+L \int \limits _{0}^{\infty } y^2 \,\mu (dy), \end{aligned}$$

where $L=2{\mathbb {E}}Z^2$. Since $0\le Z\le 1$, we have $L=2{\mathbb {E}}Z^2<2{\mathbb {E}}Z=1$.

#### *Remark 1*

The kernel $k$ in Example 3 is not a continuous function even if the density $h$ is a continuous and we cannot apply directly Theorem 4 in this case. But it not difficult to check that if $q>0$ then $\mu ^*(\{0\})=0$ and to prove that the invariant measure $\mu ^*$ has a density $u_*$, and $u_*$ is a continuous function on the interval $(0,\infty )$. Moreover, repeating the proof of Theorem 4 one can check that $\mu _t=e^{-t}\mu _0+v_t(x)\,dx$, and $v_t$ converges uniformly to $u_*$ on the sets $[\varepsilon ,\infty )$, $\varepsilon >0$. In particular, if we consider Eq. (29) on the space of probability densities, then every solution converges to $u_*$ in $L^1[0,\infty )$.

## Conclusion

In the paper we presented some phenotype structured population models with a sexual reproduction. We consider both random and assortative mating. Our starting point is the individual-based model which clearly explains all interactions between individuals. The limit passage with the number of individuals to infinity leads to the macroscopic model which is a nonlinear evolution equation. We give some conditions which guarantee the global existence of solutions, persistence of the population and convergence of its size to some stable level. Next, we consider only a population with random mating and under suitable assumptions we prove that a phenotypic profile of the population converges to a stationary profile.

It would be interesting to study analytically long-time behavior of a phenotypic profile of population with assortative mating. Some numerical results presented in the paper Doebeli et al. (2007) suggest that also in this case one can expect convergence of a phenotypic profile to multimodal limit distributions. This result suggests that assortative mating can lead to a polymorphic population and adaptive speciation. We hope that our methods invented to asymptotic analysis of populations with random mating will be also useful in the case of assortative mating. In order to do it, we probably need to modify the model of assortative mating (7) presented in Sect. 2, because it has a disadvantage that the mating rate does not satisfy the condition $\sum _{j=1}^n m(x_i,x_j)= 1$ for all $i$. We can construct a new model which corresponds to the same preference function $a(x,y)$ with a symmetric mating rate $m$ which has the above property. In order to do this we look for constants $c_1,\dots ,c_n$ depending on the state of population such that

50 $$\begin{aligned} m(x_i,x_j;{\mathbf {x}})=(c_i+c_j)a(x_i,x_j) \end{aligned}$$

and $\sum _{j=1}^n m(x_i,x_j;{\mathbf {x}})= 1$ for all $i$. In this way we obtain a system of linear equations for $c_1,\dots ,c_n$:

51 $$\begin{aligned} \sum _{j=1}^n b_{ij}c_j=1,\quad \mathrm{for}\,i=1,\dots ,n, \end{aligned}$$

where $b_{ij}=a(x_i,x_j)$ for $i\ne j$ and $b_{ii}=a(x_i,x_i)+\sum _{l=1}^n a(x_i,x_l)$. Since the matrix $[b_{ij}]$ has positive entries and the dominated main diagonal, system (51) has a unique and positive solution. The passage with the number of individuals to infinity leads to the following mating rate

52 $$\begin{aligned} m(x,y;\mu )=(c(x;\mu )+c(y;\mu ))a(x,y), \end{aligned}$$

where the function $c(x;\mu )$ depends on a phenotypic distribution $\mu$, and satisfies the following Fredholm equation of the second kind

53 $$\begin{aligned} c(x;\mu )\int \limits _F a(x,y)\,\mu (dy)+ \int \limits _F c(y;\mu )a(x,y)\,\mu (dy)=1. \end{aligned}$$

One can introduce a general model which covers both semi-random and assortative mating. Let $p(x)$ be the initial capability of mating of an individual with trait $x$ and $a(x,y)$ be a symmetric nonnegative preference function. Now we can define a *cumulative preference function* by $\bar{a}(x,y)=a(x,y)p(x)p(y)$. The mating rate $m$ is a symmetric function given by (50) with $a$ replaced by $\bar{a}$ and we assume that $\sum _{j=1}^n m(x_i,x_j;{\mathbf {x}})=p(x_i)$ for each $i,j$. The mating rate in an infinitesimal model is of the form

54 $$\begin{aligned} m(x,y;\mu )=(c(x;\mu )+c(y;\mu ))a(x,y)p(x)p(y), \end{aligned}$$

where the function $c(x;\mu )$ satisfies the following equation

55 $$\begin{aligned} c(x;\mu )\int \limits _Fa(x,y)p(y)\,\mu (dy)+ \int \limits _F c(y;\mu )a(x,y)p(y)\,\mu (dy)=1. \end{aligned}$$

In particular, in the semi-random case we have $a\equiv 1$ and $c\equiv 1/\int _F 2p(y) u(y)\,\mu (dy)$ and the mating rate is given by (3). Let us recall that in the general case $c$ is not only a function of $x$ but it is also depends on $\mu$ and therefore, the proofs of results from Sects. 3 and 4 cannot be automatically adopted to these models.
